# Transformation behavior of hazardous jarosite into recyclable hematite in a solution with high concentrations of K^+^ and Na^+^

**DOI:** 10.1038/s41598-024-64502-w

**Published:** 2024-06-17

**Authors:** Yubo Xing, Zhigan Deng, Chang Wei, Xingbin Li, Minting Li

**Affiliations:** 1 Faculty of Metallurgical and Energy Engineering, Kunming University of Science and Technology, Kunming, 650093 China; 2 State Key Laboratory of Complex Nonferrous Metal Resources Clean Utilization, Kunming University of Science and Technology, Kunming, 650093 China

**Keywords:** Simulated solution, Hematite products, Leaching solution, Concentration ratio, Molecular dynamics, Chemical engineering, Environmental chemistry

## Abstract

Iron in the leaching solution with high K^+^ and Na^+^ concentrations was usually precipitated as the typical hazardous and toxic jarosite residues. However, this method of treatment has been greatly restricted by increasingly strict environmental regulations. Here we propose that iron can be precipitated from the solution with high K^+^ and Na^+^ concentrations as recyclable hematite products by adjusting the concentration ratio of sodium and potassium ions in the solution. The transformation behavior of jarosite into hematite in high concentration potassium ion and sodium ion solution was explained based on collision theory. The results indicated that in instances where the concentration ratio of Na^+^/K^+^ is ≥ 4:1, the iron present in the solution can be effectively precipitated as a recyclable hematite product, as opposed to forming the conventional hazardous jarosite residue, even under conditions where the potassium ion concentration reaches levels as high as 4 g/L. On the other hand, thermodynamic and molecular dynamics simulations indicate that at a temperature of 185 °C, the decomposition transformation of Na-jarosite (32.64 kJ and 7.25 eV) is more energetically advantageous compared to that of K-jarosite (61.07 kJ and 15.31 eV). The results were verified by the leaching solution from smelting industry. The iron content in the residues is above 58%, the sulfur content is below 4%, the zinc content is below 1%, and the total iron concentration in the supernatant is about 4 g/L, reaching the production index of the smelting industry. The green, environmentally friendly, and recyclable separation of iron in a solution with high concentrations of potassium and sodium ions is achieved, which is of great significance for the treatment of iron-containing solution and wastewater in the chemical industry and metallurgy fields.

## Introduction

Since the end of the 1960s, to produce high-quality metal electrical products and a high current efficiency, three main processes have been developed for the removal of excess iron in the zinc hydrometallurgical industry: the jarosite (MFe_3_(SO_4_)_2_(OH)_6_, M = K^+^, Na^+^, H_3_O^+^ or NH_4_^+^) process, the goethite (α-FeOOH) process and the hematite (α-Fe_2_O_3_) process^1–6^ that are named according to the main iron compound precipitated during the process. Moreover, simple neutralization process is sometimes used. At this conditions, however, ferric ions can be hydrolyzed to form ferric hydroxide (Fe(OH)_3_) precipitates which are difficult to filter and adsorb large amounts of the associated heavy metals^7^. As a hazardous and toxic waste material, the jarosite and goethite residues need to be stored in controlled and special lined tailings sites, which exhibit the shortcomings of high occupancy space, high construction costs and high landfilling and pretreatment cost^8^. Even so, leaching of heavy metal elements and toxic elements from these residues, such as zinc, nickel, copper, cobalt, arsenic, and lead, etc., may cause contamination of groundwater and soil, resulting in a serious harm to the local ecological environment^9–11^. So far, there is no suitable processing to comprehensively recycle of such residues in industrial production. Together with the increasingly stringent global environmental regulations, among the currently available processes, iron precipitation as hematite from hydrometallurgical process solutions is the most suitable from an environmental point of view^12^.

The separation of zinc and iron by the hematite process has the following advantages. First, it offers a more environmentally friendly product because of its compact design (4.9–5.3 g/cm^3^ density) and high thermodynamic stability of hematite, which can be safely impounded^13^. Second, only 0.2 ton of dry residue is produced by the hematite process for per ton of zinc, while the jarosite process and goethite process will yield 0.5 t and 0.32 t of dry residue under the same conditions^14^. Third, the latter two processes, generated large volumes of residues, commonly contain undesirable high levels of hazardous impurities like cobalt, arsenic, and lead. The products from hematite process are low sorption of water or other impurities^5^. Fourth, the zinc content in hematite residue ranges from 0.5% to 1%, which is much lower than 6% in jarosite residue and 8% in goethite residue, thus significantly reducing the loss of valuable metal. Last, the iron content of dry basis of the iron removal residues (jarosite and goethite residues) is only 10–40% due to the high impurity content, which makes them difficult to reuse economically. The iron content (dry basis) of hematite residues is 55–65%, which is similar to the iron content in pure hematite (69.94%), and a marketable product can be used as a reagent in cement, pigment, and, potentially, in the steel making industries^15–17^. The Iijima Zinc Refinery (Akita, Japan) and Yunnan Hualian Zinc and Indium Co., Ltd (Kunming, China) successfully applied the hematite process to industry in 1972 and 2018, respectively, and sold all hematite residues to the cement manufacturing industry and steel making industry, realizing comprehensive recovery of valuable metals and zero-emission for a pollution-free environment^14,18^.

Some developments, as practiced in the zinc industry, have been made in the past decade from studies on the iron removal by the hematite process. The chemistry of this process has been described extensively in the literature^19–23^. In the absence of other ions, the hematite process involves the oxydrolysis reaction of ferrous sulfate is a sum of two reaction steps:

oxidation of ferrous sulfate to ferric sulfate:

1 $$4{\text{FeSO}}_{4} + 2{\text{H}}_{2} {\text{SO}}_{4} + {\text{O}}_{2} \left( {\text{g}} \right) = 2{\text{Fe}}_{2} \left( {{\text{SO}}_{4} } \right)_{3} + {\text{2H}}_{2} {\text{O}}$$

and hydrolysis of ferric sulfate to produce hematite:

2 $${\text{Fe}}_{2} \left( {{\text{SO}}_{4} } \right)_{3} + 3{\text{H}}_{2} {\text{O}} = {\text{Fe}}_{2} {\text{O}}_{3} + {\text{3H}}_{2} {\text{SO}}_{4}$$

The hydrolytic reaction of ferric ions becomes complicated under hydrothermal conditions when the presence of impurity ions in the solution, such as Na^+^, K^+^, NH_4_^+^, etc. During the reaction, ferric ions can be hydrolyzed to form jarosite precipitates:

3 $${\text{Na}}_{2} {\text{SO}}_{4} + 3{\text{Fe}}_{2} \left( {{\text{SO}}_{4} } \right)_{3} + 12{\text{H}}_{2} {\text{O}} = 2{\text{NaFe}}_{3} \left( {{\text{SO}}_{4} } \right)_{2} \left( {{\text{OH}}} \right)_{6} + {\text{6H}}_{2} {\text{SO}}_{4}$$

The Na^+^ ion in Eq. (3) can be replaced by K^+^ or NH_4_^+^ to form jarosite. Previous studies have shown that sodium jarosite is readily converted into hematite by hydrothermal reaction^23^. The conversion of jarosite to hematite is illustrated by Eq. (4).

4 $$2{\text{NaFe}}_{3} \left( {{\text{SO}}_{{4}} } \right)_{2} \left( {{\text{OH}}} \right)_{6} = {\text{Na}}_{2} {\text{SO}}_{4} + 3{\text{Fe}}_{2} {\text{O}}_{3} + 3{\text{H}}_{2} {\text{SO}}_{4} + {\text{3H}}_{2} {\text{O}}$$

The effects of several physicochemical variables and reaction kinetics have been elucidated, and the effects of different operating conditions on the composition and action of K, Na, Cu, and Zn have been investigated^12,17^. The purpose of these studies has been to determine the important parameters for the design, operation, and optimisation of plant performance of the hematite process.

In previous work, most of the investigations of the hematite process have been carried out based on low concentrations and single variates of K, Na, and Zn^14,24^. The concentration of sodium ions and potassium ions in the solution has a critical influence on whether the iron in the solution is finally separated by precipitation in the form of the typical hazardous and toxic jarosite residues or recycled hematite products. In the smelting process to recover zinc-containing waste, the leaching solution of secondary zinc oxide powder (SZOP) from combined pyro-hydrometallurgical processes of iron and steelmaking dust and sludge^25^ contains a high concentration of zinc, potassium ions and sodium ions. Obviously, all the above studies on hematite process could not effectively solve the separation problem of iron in the leaching solution of SZOP. The green and efficient separation of iron in the leaching solution of the SZOP has become an urgent problem to be solved in practical production.

Therefore, the hematite process of the solution with high concentrations of potassium ions and sodium ions under hydrothermal conditions were studied, focusing on the effects of the concentrations of potassium ions, sodium ions and their concentration ratio on the contents of iron, sulfur, potassium, and sodium in the iron removal residues, as well as the iron concentration in the supernatant after iron removal. Then, this behavioral law was verified in a leaching solution, proving the feasibility of removing iron by hematite process in the leaching solution of the SZOP. The corresponding decomposition mechanism of jarosite crystals were derived by quantum chemical study. Finally, we can separate iron from a solution with high concentrations of potassium and sodium ions into the form of hematite, which can be used as a resource. This method provides an effective solution to the environmental harm and the difficulties in recycling iron residues resources caused by iron removal via the jarosite process.

## Materials and methods

### Apparatus and materials

A Büchiglas-eloclave (49.32232 polyclave Type 2.0 It.) was used as the reaction vessel for the precipitation tests. The autoclave was equipped with a heating circulation bath, a sampling system, a proportional-integral–differential temperature controller, data logging software on a personal computer, and a stirrer with a rotation speed of 0–1500 rpm. The working temperature of the autoclave was set in the range of 0–300 °C to meet the requirements of the hematite process.

Analytical grade K_2_SO_4_, Na_2_SO_4_, FeSO_4_⋅7H_2_O, ZnSO_4_⋅7H_2_O, concentrated H_2_SO_4_ (98%) and deionized water were used to prepare the initial solutions. All chemicals were of reagent grade, obtained from commercial sources, and used without further purification. An industrial feed solution with pH 4.68, obtained from the leaching solution of the SZOP after the indium precipitation process, was provided by a clean utilization and harmless treatment of the heavy metal waste plant (Xin Lian GreenNovo Environmental Technology Co., Ltd., Honghe, China).

### Experimental procedures

Based on the hematite process parameters of the Yunnan Hualian Zinc and Indium Co., Ltd, Kunming, China, the Iijima hematite plant, and our previous research results^12^, the experimental conditions were as follows: a total reaction duration of 3 h, an oxygen partial pressure of 0.4 MPa, a seed addition of 15 g/L, a reaction temperature of 185 °C, and an agitation speed of 500 rpm.

**Figure 1:**
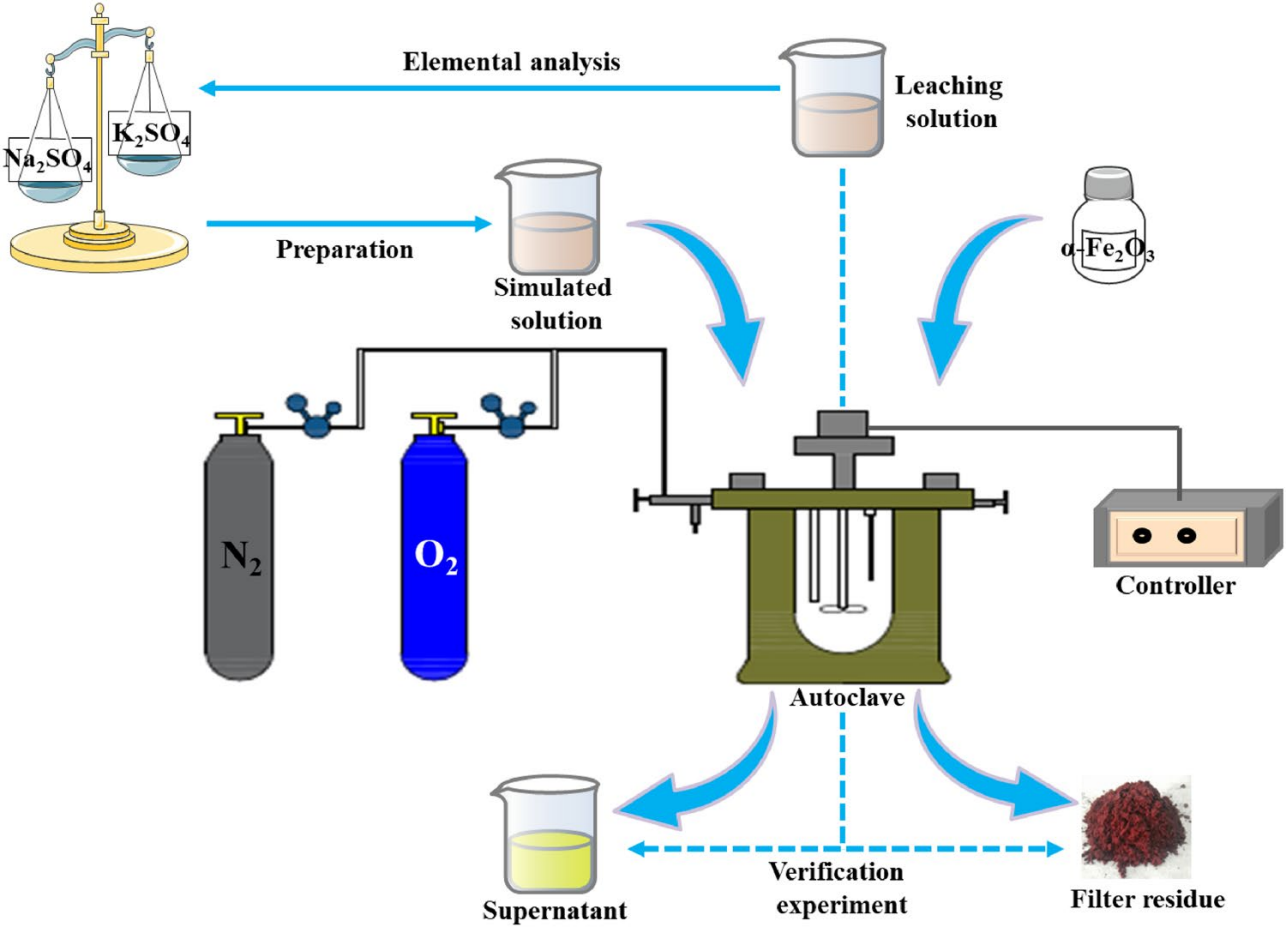
Schematic diagram of the experiment.

According to the composition of industrial feed solution, the concentration of each ion in the simulated solution were as follows: 135 g/L of zinc ions, 25 g/L of total iron ions, potassium ions range of 0–5 g/L, and sodium ions range of 0–25 g/L. The concentration ratio of Na^+^/K^+^ were 0 (no these two ions), 1:1, 2:1, 3:1, 4:1, and 5:1, respectively. The procedures of hematite process experiments are shown in Fig. 1. Before each experiment, the autoclave was carefully cleaned. Then, 1 L of the simulated solution or the industrial feed solution and a certain amount of seed was transferred into the autoclave. In the process of heating to the set temperature, nitrogen gas (purity ≥ 99.2%) was introduced into the autoclave several times using nitrogen supply equipment to obtain a complete anaerobic atmosphere. When the temperature reached the target temperature, oxygen was introduced, and timing was started. The oxygen partial pressure was fixed to the desired level and maintained at a constant value throughout the experiment. After the end of the reaction, agitation was stopped, and the reaction solution in the autoclave was rapidly cooled by a cooling coil through a tray cold tube. The slurry was filtered with a vacuum pump and a Buchner funnel when the temperature of the slurry decreased to 70 °C. The filter residues were thoroughly washed with water and then dried in an oven at 55 °C for at least 24 h prior to analysis.

### Analytical techniques

The concentration of iron in the solution was determined by potassium dichromate titration. The pH of the solution was measured using a temperature compensation pH meter (S220-K-CN, METTLER TOLEDO, Switzerland). The filter residues were characterized by X-ray diffraction (XRD; CuKα radiation, D/Max 2200, Rigaku, Japan) to determine their phase composition. The zinc content in the filter residues was determined using atomic absorption spectroscopy (ASS). The solids were chemically analyzed to determine their Fe content. The sulfur content in the filter residues was detected by the infrared absorption method after combustion in the induction furnace. Inductively coupled plasma-optical emission spectrometry (ICP-OES, iCAP 7000, Thermo Fisher Scientific, UK) was used to determine the K and Na contents in the precipitation products. Scanning electron microscopy (SEM, Gemini 300, ZEISS, Germany) was used to study the microstructure and surface composition of the iron precipitates. Fourier transform infrared (FT-IR) spectroscopy was used to confirm the sulfate ions and their peak intensity in the range of 400 to 4000 cm^−1^.

### Computational details of quantum chemical

The quantum chemical calculations for isolated Na-jarosite and K-jarosite were performed to all the calculations in the paper by the first-principles density functional theory (DFT) using the Cambridge Serial Total Energy Package (CASTEP) method and the Materials Studio 8.0 program. For the geometry optimization of cell and supercell structure, the Perdew-Burke-Ernzerh of (PBE) form of Generalized Gradient Approximation (GGA) and Broyden–Fletcher–Goldfarb–Shanno (BFGS) algorithm was adopted. The atomic relaxation convergence was set to 0.05 eV/Å, the energy convergence was set to 2.0 × 10^–5^ eV/atom, the electronic Self-consistent Field (SCF) convergence was set to 2.0 × 10^–6^ eV/atom, the root-mean-square atomic displacement was set to 0.002 Å, and the cut-off energy of plane wave basis was 330 eV. The 2 × 2 × 1 Monkhorst–Pack grid was used as the Brillouin zone k-points during the relaxation.

After the geometric optimization of supercell structure, the molecular dynamics of it was calculated by the Perdew-Burke-Ernzerh of (PBE) form of Generalized Gradient Approximation (GGA) under the NVT (fixed number of particles, volume and temperature) ensemble by the Nosé-thermostat. The number of steps was 1000, the temperature was set to 458.15 K, the time step was 1 fs, and the simulation time was 1 ps. A plane-wave basis set with kinetic energy cutoff of 330 eV was used. Integration over the Brillouin zone was performed by using the Gamma-centered Monkhorst–Pack scheme with (2 × 3 × 1) k-points.

The energies of jarosite supercell decomposition (*E*_*decomposition*_) were calculated using the following equation:

5 $$E_{decomposition} = E_{dynamics} - E_{optimization}$$

where *E *_*dynamics*_ and *E *_*optimization*_ are the energies of the molecular dynamics of jarosite supercell and it after optimization.

## Results and discussion

### The hematite process of simulated solution

#### Effect of potassium ion concentrations

The hazardous and toxic waste material of K-jarosite, which is difficult to decompose at this process temperature, significantly impacts the iron and sulfur content in the hematite product. The hematite process of the simulated solution was assessed for various initial concentrations of potassium ions in the range of 0 g/L to 5 g/L, which were adjusted using K_2_SO_4_ of analytical grade.

**Figure 2:**
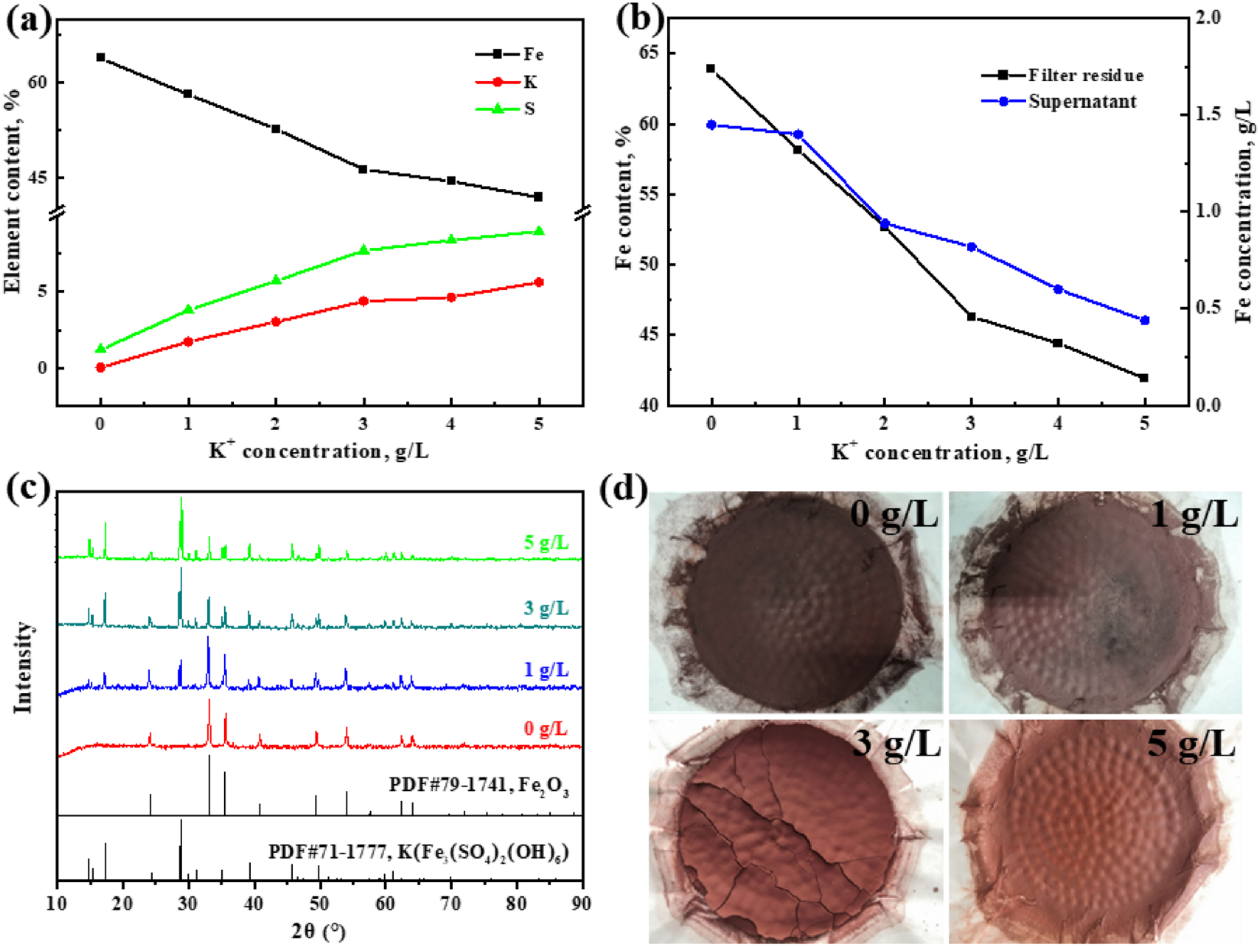
Effect of potassium ion concentration on iron, sulfur and potassium content of the filter residues (**a**) and total iron concentration of the supernatant after iron removal (**b**); XRD patterns of the filter residues at different potassium ion concentration (**c**); the images of the filter residues at different potassium ion concentrations (**d**).

The elemental content of the filter residues and supernatant after iron removal by the hematite process is depicted in Fig. 2, specifically focusing on the concentrations of potassium ions. As evidenced by the results, the iron content in both the filter residues and supernatant decreased as the concentration of potassium ions increased, with ranges of 63.86% to 41.91% and 1.45 g/L to 0.44 g/L (Fig. 2b), respectively. In contrast, the K and S content in the filter residues showed the opposite trend and increased from 0 to 5.64% and 1.2% to 8.98% (Fig. 2a), respectively. The process index of hematite in the field of chemical industry and metallurgy is that the iron content in the residue is above 55%, the sulfur content is below 5%, the zinc content is below 1%, and the total iron concentration in the supernatant is about 3 ~ 5 g/L^26,27^. Therefore, to obtain high-quality hematite residues, the maximum concentration of potassium ions allowed to exist alone in the solution is 1 g/L.

In order to elucidate the impact of potassium ions on the residue phase during the hematite processing, the filter residues collected under varying potassium ion concentrations were subjected to X-ray diffraction analysis. The obtained XRD patterns are presented in Fig. 2c. The XRD analysis indicates that in the absence of potassium ions in the solution, only hematite was identified in the filter residues. The purity of the residue is high, with no discernible impurity peaks detected. By incrementally increasing the potassium ion concentration from 0 g/L to 5 g/L, the distinct peaks corresponding to K-jarosite became more pronounced and well-defined in the XRD pattern. As a result, the higher the potassium ion concentration, the lower the iron content in both the supernatant and the filter residues, attributable to the formation of K-jarosite. As illustrated in Fig. 2d, it is evident that the color of the residue gradually changes from a deep red to a light red with a slight yellowish hue due to the formation of K-jarosite. This observation is consistent with the results of elemental analysis and XRD analysis.

**Figure 3:**
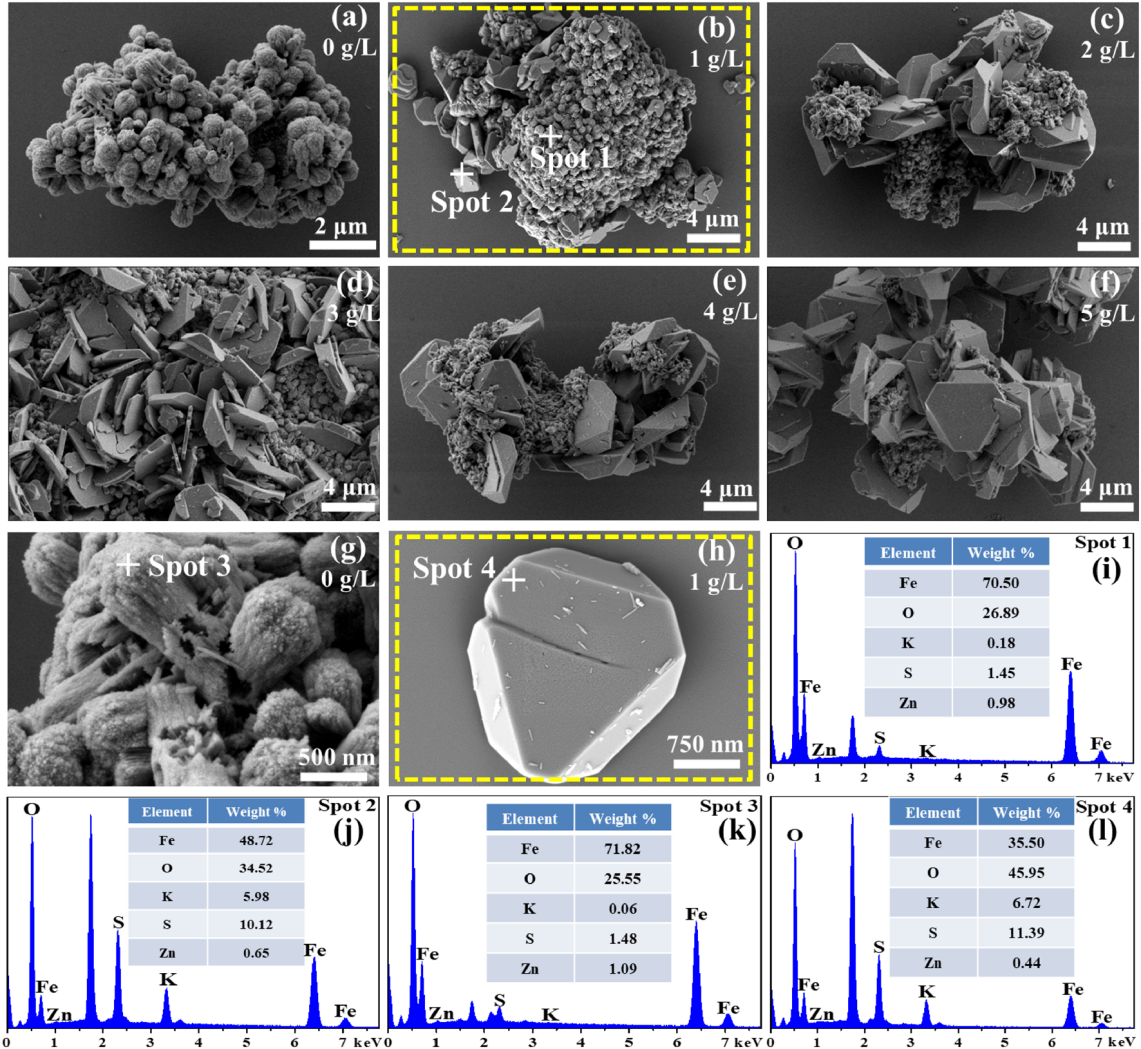
SEM images and EDS analysis of the filter residues at different potassium ion concentrations.

**Figure 4:**
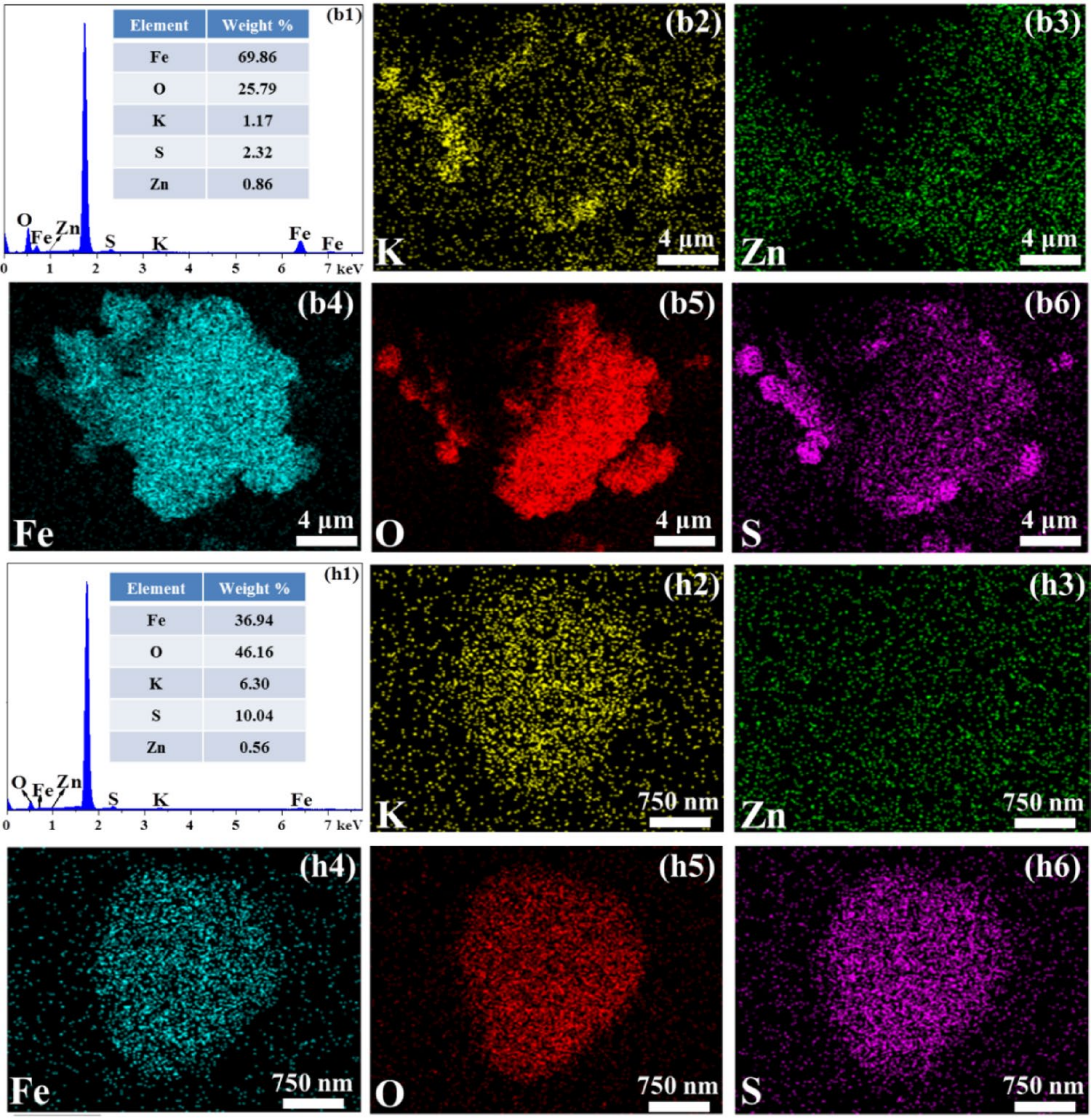
The EDS analysis and element distribution maps of the filter residues at 1 g/L potassium ion (**b1**–**b6**) and the hexagonal block-shaped particles in filter residues (**h1**–**h6**).

The morphology of the filter residues formed at various concentrations of potassium ions was examined using SEM, which served to confirm the overall structure and distribution of elements. The SEM images of the filter residues and the chemical composition of the selected particle are presented in Figs. 3 and 4. The irregularly-shaped particles, which appeared to be aggregates of small spherical crystals (Fig. 3a,b, and g), were identified as hematite based on their high Fe and O contents and low K and S contents (Fig. 3i, and k). The other type comprises dispersed hexagonal block-shaped particles (Fig. 3b–f, and h) with a large volume, regular structure, and relatively smooth surface. The EDS (Fig. 3j, and l) and elements mapping analysis (Fig. 4h1–h6) selected the area in Fig. 3(h) as K-jarosite due to a large proportion of K, S, and O, and low Fe content. When the concentration of potassium ions is ≤ 1 g/L, it can be observed from the elements mapping analysis (Fig. 4b1–b6) of the selected area in Fig. 3a,b that the main phase in the residue is hematite. As the concentration of potassium ions increases, the hexagonal block-shaped particles in the filter residues gradually increase, and the primary phase of the filter residues gradually changes from hematite to K-jarosite phase. This further demonstrates the significant impact of potassium ion concentration in the solution on the phase composition of iron precipitates.

#### Effect of sodium ion concentrations

**Figure 5:**
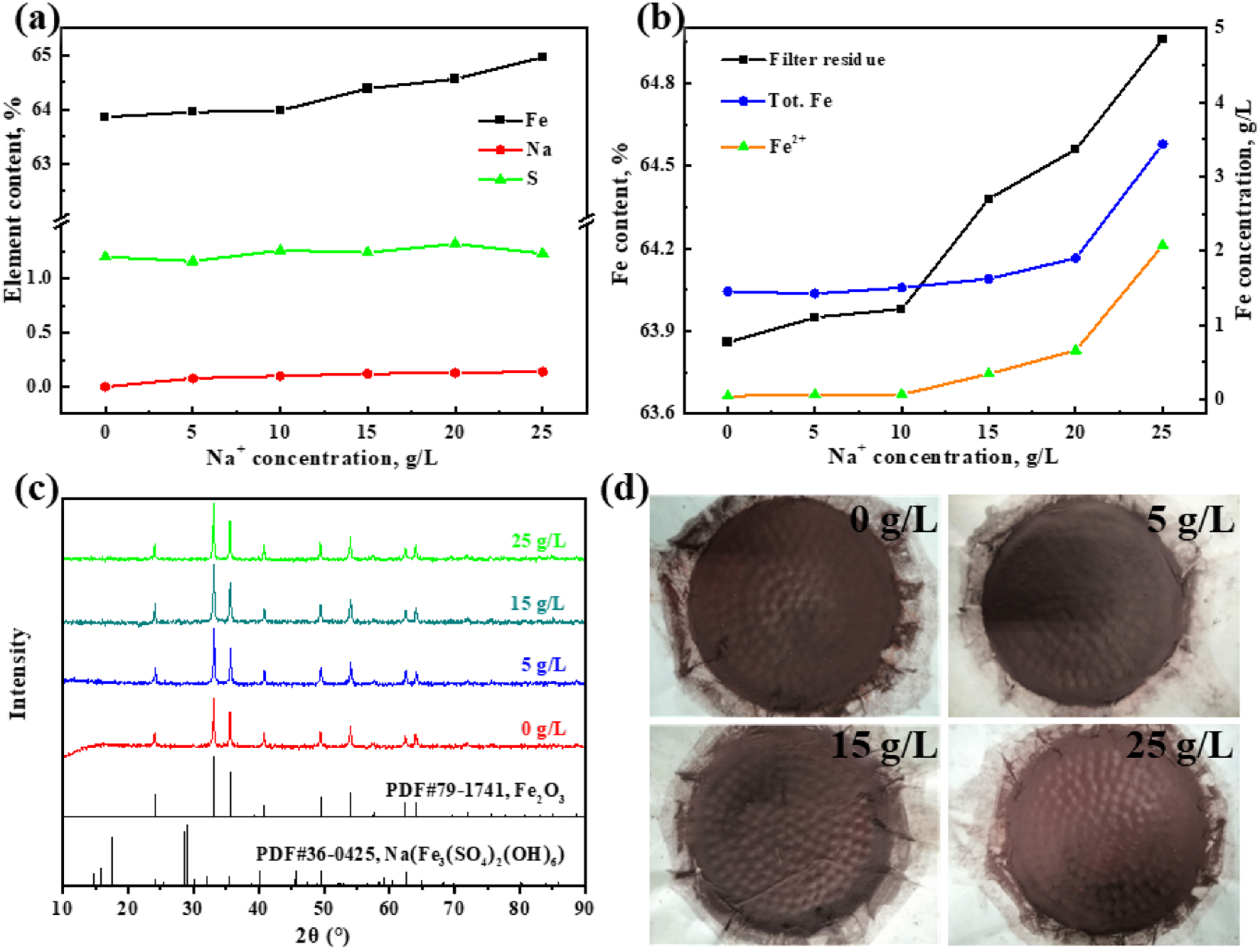
Effect of sodium ion concentration on iron, sulfur and potassium content of the filter residues (**a**) and total iron concentration of the supernatant after iron removal (**b**); XRD patterns of the filter residues at different sodium ion concentrations (**c**); the images of the filter residues at different sodium ion concentrations (**d**).

The effects of sodium ion concentrations on the contents of iron, sulfur, and sodium in the filter residues, as well as the iron concentration in the supernatant after iron removal, were tested using various detection methods. The results are shown in Fig. 5. When the sodium ion concentration was increased from 0 g/L to 25 g/L, the content of Fe, Na, and S in the filter residues only increased by 1.1%, 0.14%, and 0.16%, respectively (Fig. 5a). Hence, the impact of sodium ions on the quality of the filter residue is deemed insignificant. Nonetheless, the total iron concentration in the supernatant exhibited a clear increase with varying sodium ion concentrations, with a range of variation from 1.45 g/L to 3.43 g/L as illustrated in Fig. 5b. The dissolution and crystallization of ferrous sulfate is carried out throughout the duration of the hematite process. The introduction of sodium ions results in a large quantity of sulfate ions being introduced into the solution, causing the reaction (Eq. 6) to shift to the left (towards the reactants). The solubility of ferrous sulfate is consequently decreased. After the reaction is complete, with the decrease in temperature, the undissolved ferrous sulfate re-dissolves into the supernatant, leading to an increase in the concentration of ferrous ions in the solution.

6 $${\text{FeSO}}_{{4}} \cdot{\text{H}}_{{2}} {\text{O}}\left( {\text{s}} \right) = {\text{Fe}}^{2 + } + {\text{SO}}_{4}^{2 - } + {\text{H}}_{2} {\text{O}}$$

The filter residues obtained at different concentrations of sodium ions were characterized by XRD, with the results shown in Fig. 5c. The characteristic diffraction peaks associated with the Na-jarosite phase were not observed in the residues. It is evident that the predominant component of the filter residues was hematite, and variations in the sodium ion concentration did not significantly alter the composition of the filter residues. Furthermore, as depicted in Fig. 5d, there was no noticeable alteration in the color of the residues.

**Figure 6:**
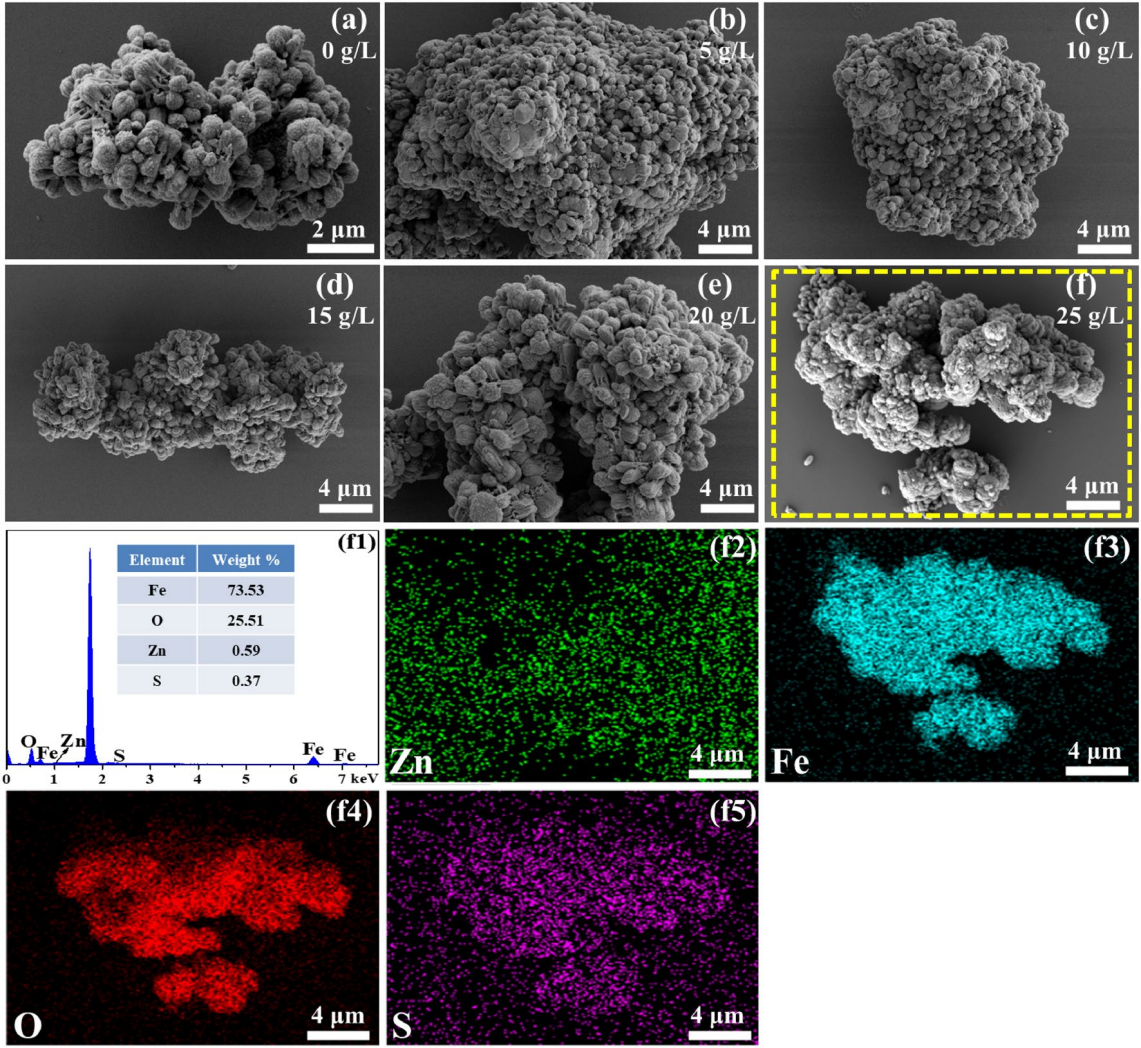
The SEM images of the filter residues at different sodium ion concentration (**a**–**f**) and element distribution maps at 25 g/L sodium ion (**f**–**f5**).

The morphology, EDS analysis, and element distribution maps of the filter residues were investigated using scanning electron microscopy (SEM), as illustrated in Fig. 6. The SEM images reveal that the introduction of sodium ions has no obvious effect on the morphology of the hematite residues. The hematite primarily exists in the form of irregular agglomerates comprising nano-scale small particles. The elemental mapping analysis of the filter residues indicated that the distribution of Fe, O, and S is relatively concentrated. Zinc is adsorbed, mixed, and encapsulated in the residue in the form of zinc sulfate, and it is evenly distributed on the silicon wafer after being dispersed by an alcohol dispersant. This observation is in accordance with the findings of elemental analysis and X-ray diffraction analysis.

#### Effect of the concentration ratios of Na^+^/K^+^

**Figure 7:**
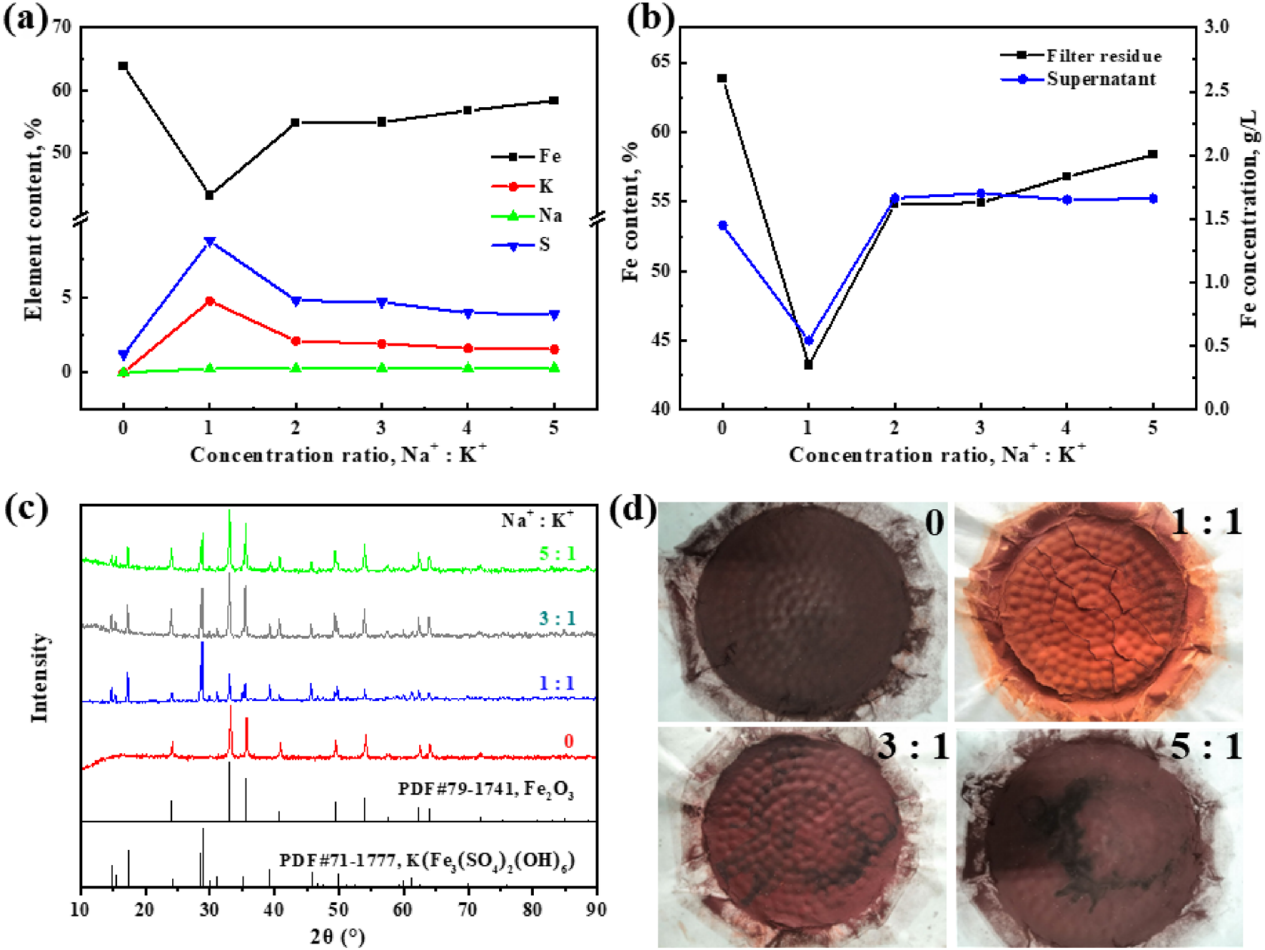
Effects of different concentration ratios of Na^+^/K^+^ on the contents of iron, sulfur, potassium and sodium in the filter residues (**a**) and total iron concentration of the supernatant after iron removal (**b**); XRD patterns (**c**) and the images of the filter residues with different concentration ratios of Na^+^/K^+^ (**d**).

In practical industrial applications, the leaching solution of SZOP typically simultaneously contains high concentrations of potassium ions and sodium ions in the pyro-hydrometallurgical processes of iron and steelmaking dust and sludge. In this study, conducted for the purpose of producing high-quality hematite products, the influence of the Na^+^/K^+^ concentration ratio on the iron, sulfur, potassium, and sodium contents in filter residue was investigated for the first time. Additionally, the impact of the iron concentration in the supernatant after iron removal was examined, as illustrated in Fig. 7.

The initial potassium ion concentration in the simulated solution was fixed at 4 g/L. The solution was then studied with varying initial sodium ion concentrations ranging from 4 g/L to 20 g/L, achieved by adjusting the concentration using analytical grade Na_2_SO_4_. As mentioned earlier, in the absence of sodium and potassium ions in the solution, the filter residue phase mainly consisted of hematite, with the iron content in the residue being 63.86% (Fig. 7a), which is similar to the iron content in pure hematite (69.94%). The analysis of Fig. 7a indicates that with the increase in the Na^+^/K^+^ concentration ratio, there is a significant increase in the iron content within the filter residues, with a variation range of 43.23% to 58.34%. By contrast, the K and S content in the filter residues exhibited an opposite trend, decreasing from 8.76% to 3.88% for K and from 4.76% to 1.54% for S, respectively, indicating a gradual reduction of the K-jarosite phase in the residues. On the other hand, as depicted in Fig. 7b, with a Na^+^/K^+^ concentration ratio of 1:1, the total iron concentration in the supernatant drastically decreased to 0.546 g/L owing to the substantial formation of K-jarosite. The sodium content in the residues remains stable at about 0.28%, indicating that the conversion of Na-jarosite in the hematite process is complete. It was found that the concentration ratio of sodium and potassium ions in the solution played a crucial role in the process of the transformation of the jarosite residues into the hematite products.

To further substantiate the aforementioned findings, X-ray Diffraction (XRD) analysis was employed to analyze the precipitates, as illustrated in Fig. 7c. It can be observed that only hematite was detected in the filter residues when the solution lacked sodium and potassium ions. When the concentration ratio of Na^+^/K^+^ is 1:1, the diffraction peak of K-jarosite is stronger in the sample, indicating that K-jarosite is the main component in the formed precipitate, leading to higher potassium (8.76%) and sulfur (4.76%) content in the precipitate. As shown in Fig. 7d, it is clear that the color of the residues is yellow at 1:1 of the concentration ratio of Na^+^/K^+^, which is the characteristic color of jarosite. As the concentration ratio of Na^+^/K^+^ increases, the characteristic peak of K-jarosite in the precipitation gradually weakens, while that of hematite gradually increases. This leads to an increase in iron content and a decrease in potassium and sulfur content in the precipitation. When the concentration ratio of Na^+^/K^+^ was ≥ 4:1, the iron content in the filter residues increased to more than 55%, and the sulfur content decreased to less than 4%, which meets the actual production indexes of the current industrial hematite process^26,27^.

Based on elemental analysis and XRD test results, it is evident that with the increase in sodium ion concentration in the solution, the K-jarosite phase in the precipitation decreases, while the grade of hematite increases. It can be concluded from the collision theory that the higher the concentration of sodium ions in the solution, the greater the collision frequency with ferric ions, facilitating the formation of Na-jarosite. Conversely, within the effective space, the collision frequency between potassium ions and ferric ions is reduced, thereby inhibiting the formation of K-jarosite. According to the above analysis, from the analysis above, it is evident that the concentration of sodium ions has no significant effect on the composition of the filter residues. This is because, under these reaction conditions, Na-jarosite is easily decomposed into hematite.

**Figure 8:**
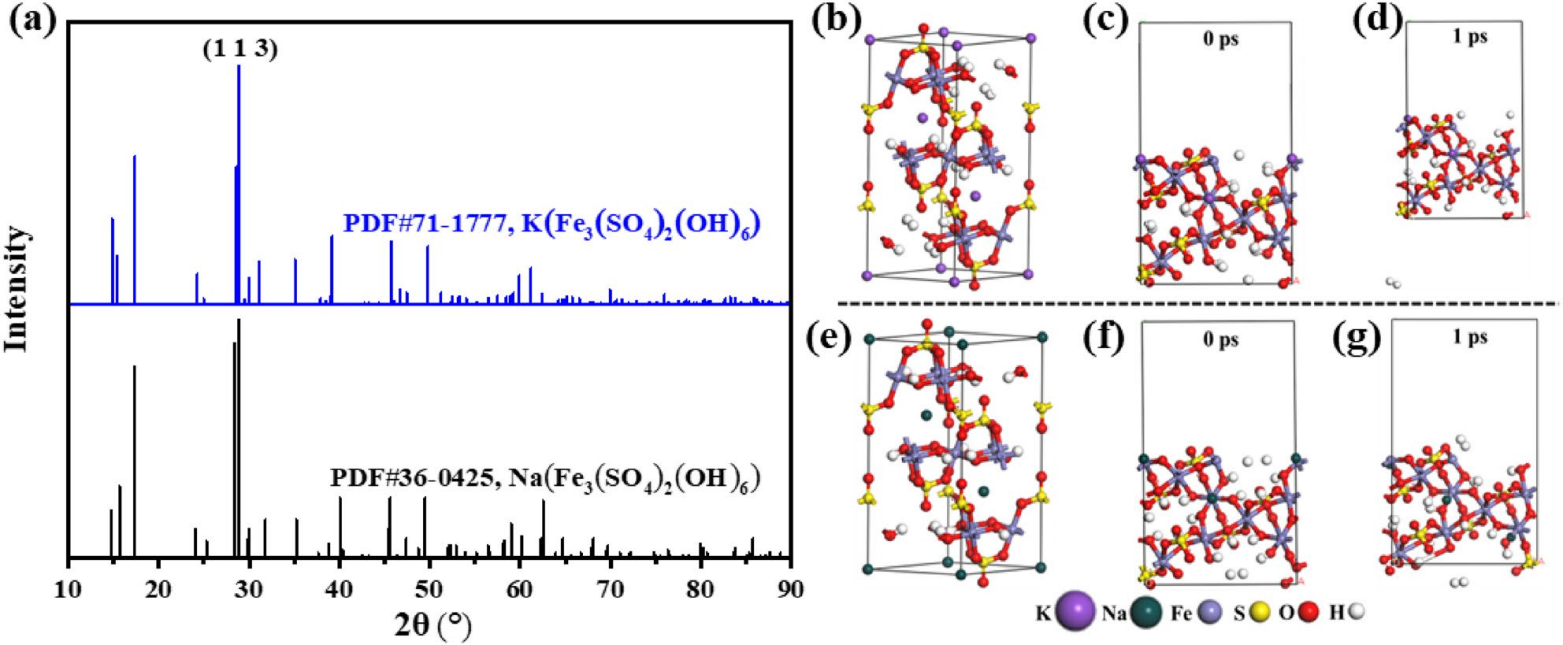
XRD patterns obtained from the MDI Jade 6.0 software (**a**); molecular structure after CASTEP geometric optimization of K-jarosite cell (**b**) and supercell (**c**) and Na-jarosite cell (**e**) and supercell (**f**); molecular structure after dynamics simulations of K-jarosite supercell (**d**) and Na-jarosite supercell (**g**).

To elucidate the decomposition behavior of jarosite, further comprehensive investigations were undertaken utilizing both molecular dynamics simulations and thermodynamic analyses. These investigations were carried out employing the computational tools CASTEP and HSC Chemistry 8.1^28^. The optimized configurations of the K-jarosite and Na-jarosite cells are depicted in Fig. 8. The results show that the optimized cell constants were a = 7.12 Å, b = 7.12 Å and c = 18.08 Å for K-jarosite (Fig. 8b), and a = 7.30 Å, b = 7.30 Å and c = 16.8 Å for Na-jarosite (Fig. 8e), which are in good agreement with experimental value^29,30^. The unit cells of K-jarosite and Na-jarosite after optimization were selected, and they were cleaved in the (1 1 3) direction for further research. This decision was based on their XRD patterns analyzed using MDI Jade 6.0 software, which indicated that the diffraction peak was strongest at the (1 1 3) plane (Fig. 8a). The dynamics model was constructed as the 1 × 1 supercell, and the thickness of surface box was set to 3. A vacuum slab of 10 Å was added to form 3D periodic boundary conditions to avoid the interaction across the mirror image in the z direction. Then, the size of the supercell will be expanded to (12.48 × 7.01 × 20.30) Å^3^. The supercell models resulting from the molecular dynamics calculations are also depicted in Fig. 8c and f. Table 1 summarizes the calculated decomposition data of K-jarosite and Na-jarosite using both HSC Chemistry 8.1 and CASTEP software.

**Table 1:** The calculated decomposition data of the Na-jarosite and K-jarosite.

Calculation method	Type	K-jarosite	Na-jarosite
Thermodynamics	*ΔG* (kJ)	61.07	32.64
	*ΔH* (kJ)	250.03	217.38
Quantum chemical	*E*_*optimization*_ (eV)	− 30,415.64	− 31,987.00
	*E*_*dynamics*_ (eV)	− 30,400.33	− 31,979.75
	*E*_*decomposition*_ (eV)	15.31	7.25

The thermodynamic calculation results indicate that the decomposed energies *ΔG* and *ΔH* of Na-jarosite at 185 °C are 32.64 and 217.38 kJ (Eq. 4), which are both lower than the values of 61.07 and 250.03 kJ for K-jarosite (Eq. 7), respectively. This indicates that the decomposition reaction of Na-jarosite is more favorable than that of K-jarosite. It can be seen from Fig. 8c and f that the 1 × 1 supercell model undergoes a significant deformation and decomposition. The result, after 1 ps dynamics simulation, indicated that hydrogen atoms on some hydroxyl groups are dissociated out of the supercell due to the breaking of O–H bonds. During the dynamics simulation, the energies of Na-jarosite and K-jarosite supercell decomposition (*E*_*decomposition*_) were 7.25 and 15.31 eV at 185 °C, indicating that Na-jarosite is more easily decomposed among the two. It can be observed that the thermodynamic analysis results regarding the decomposition order of Na-jarosite and K-jarosite are consistent with those obtained from molecular dynamics simulations.

7 $${\text{2KFe}}_{3} \left( {{\text{SO}}_{4} } \right)_{2} \left( {{\text{OH}}} \right)_{6} = {\text{K}}_{2} {\text{SO}}_{4} + 3{\text{Fe}}_{2} {\text{O}}_{3} + 3{\text{H}}_{2} {\text{SO}}_{4} + 3{\text{H}}_{2} {\text{O}}$$

### Verification experiment of hematite process in leaching solution

In combined pyro-hydrometallurgical processes of iron and steelmaking dust and sludge, SZOP leaching solution contains high concentrations of potassium and sodium ions simultaneously. In order to prevent the formation of harmful jarosite, smelting companies have been dedicated to developing environmentally friendly and recyclable methods for separating iron. To verify the above conclusions, the leaching solutions of SZOP were used to separate zinc and iron by hematite process. These solutions were provided by a clean utilization and harmless treatment of the heavy metal waste plant (Xin Lian GreenNovo Environmental Technology Co., Ltd., Honghe, China) from different production cycles, and the main components of them shown in Table 2.

**Table 2:** The main components of the leaching solution from industry.

Leaching solution number	Composition (g/L)				Concentration ratio (Na^+^ : K^+^)
	Zn	Fe	K	Na	
1	135.51	24.50	4.78	19.96	4.18
2	127.46	22.21	4.06	20.17	4.97
3	131.31	24.74	4.36	18.03	4.14

**Figure 9:**
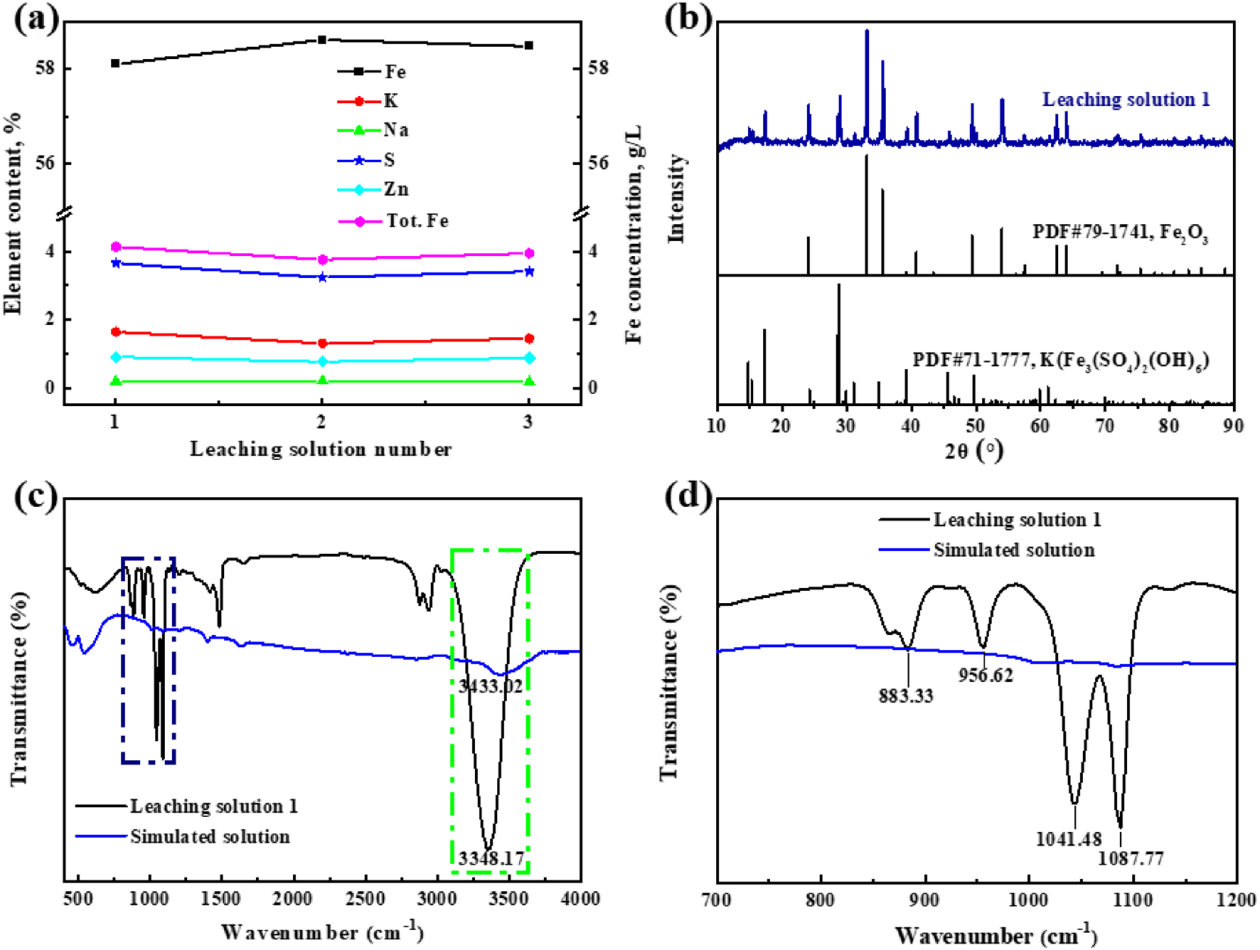
The content of iron, zinc, sodium, potassium, and sulfur in the filter residues and total iron concentration of the supernatant after iron removal under different components of leaching solution (**a**); XRD pattern of the filter residues of leaching solution 1 after iron removal (**b**); FT-IR spectra of the filter residues of leaching solution 1 after iron removal and the simulated solution in the absence of sodium and potassium ions (**c**), and enlarged view of the selected part in FT-IR spectra (**d**).

After removing iron, the contents of iron, zinc, sodium, potassium, and sulfur in the filter residues, as well as the total iron concentration of the supernatant obtained from leaching solutions with different components, are illustrated in Fig. 9a. It was found that the content of different elements in the filter residues and supernatant after the reaction is relatively stable. The iron content in the residues is above 58%, the sulfur content is below 4%, the zinc content is below 1%, and the total iron concentration in the supernatant is about 4 g/L. According to the hematite process parameters of Yunnan Hualian Zinc and Indium Co., Ltd. in Kunming, China, and the Iijima hematite plant^26,27^, these experimental results meet the production requirements of the hematite process (Table 3). Due to the high concentration of potassium ions in the leaching solution (Table 2), the formation of K-jarosite is inevitable; however, this does not affect the quality of the iron removal residues. Three groups of real solution verification experiments show that when the concentration ratio of Na^+^/K^+^ in the solution is ≥ 4:1, the hematite process can be used to separate zinc and iron in a solution containing high concentrations of sodium ions and potassium ions. It is of great significance for the treatment of iron-containing solution and wastewater in the chemical industry and metallurgy fields.

**Table 3:** Comparison of iron removal indicators in different plants.

Plant	Temperature (°C)	Precipitate (%)					Supernatant (g/L)	References
		Fe	K	Na	S	Zn	Tot. Fe	
Iijima	185	56.00	–	–	4.57	0.816	4.46	^26^
Hualian	185	58.66	0.028	0.061	2.96	1.03	3 ~ 5	^27^
Xinlian	185	58.11	1.64	0.19	3.65	0.91	4.13	Our work
		58.63	1.31	0.21	3.23	0.77	3.75	
		58.49	1.45	0.18	3.41	0.88	3.94	

The filter residues of leaching solution 1 after iron removal were characterized by XRD, and the results are shown in Fig. 9b. It was found that the filter residue was mainly composed by hematite with a minor amount of K-jarosite. Figure 9c and d shows FTIR spectrums of this residue in the range 400–4000 cm^−1^. The absorption bands at 883.33 cm^−1^, 956.62 cm^−1^, 1041.48 cm^−1^, and 1087.77 cm^−1^ can be assigned to the vibration mode of ${\text{SO}}_{{4}}^{2 - }$^31^. In addition, the absorption peaks at 3348.17 cm^−1^ and 3433.02 cm^−1^ are for the hydroxyl group (-OH)^32^. The FTIR results indicate the formation of jarosite in the present experiment, which are in agreement with the XRD measurement (Fig. 9b).

**Figure 10:**
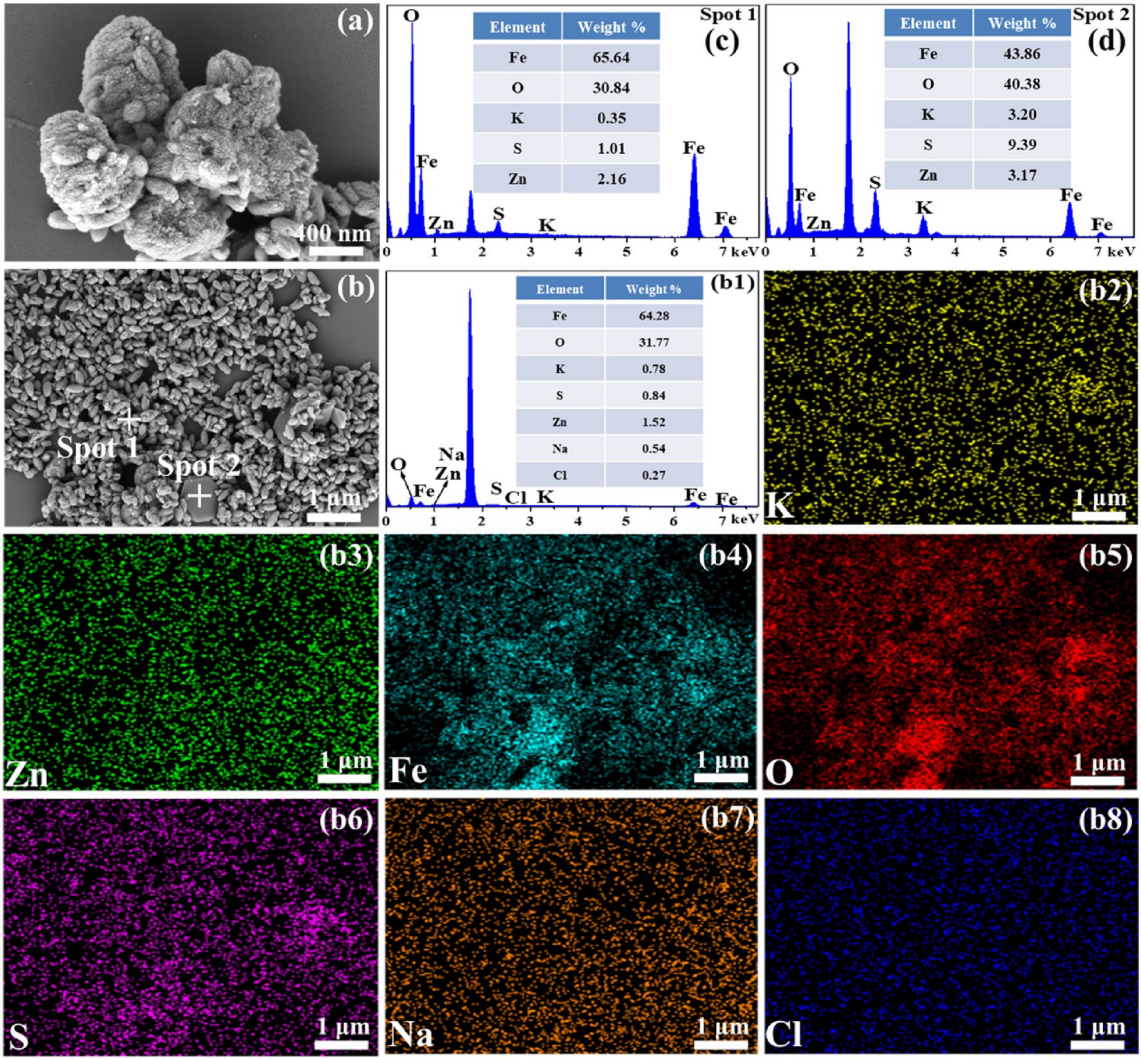
SEM images, EDS analysis and element distribution maps of the filter residues of leaching solution 1 after iron removal.

Figure 10 shows SEM image, EDS analysis and elements mapping of the filter residues of leaching solution 1 after iron removal. The filter residue consisted of smaller spindle-shaped crystals appeared to be aggregates of small spherical crystals (Fig. 10a) and larger hexagonal block-shaped particles (Fig. 10b). The main phase for the aggregated particles with high Fe and O, and low S contents can be attributed to the hematite (Fig. 10c). The block-shaped particle (Fig. 10d) is identified as K-jarosite due to a large proportion of K, S and O, and low Fe content. The SEM–EDS results of leaching solution are consistent with the simulated solution (Figs. 3 and 4).

## Conclusions

The separation of iron as recyclable hematite products from solutions characterized by high concentrations of potassium and sodium ions was successfully performed without changing the production conditions. To obtain high-quality hematite products, the maximum concentration of potassium ions allowed to exist alone in the solution is 1 g/L. Sodium ions was introduced individually have no significant effect on the composition of hematite products, even at concentrations as high as 25 g/L. When the concentration ratio of Na^+^/K^+^ is ≥ 4:1, the iron present in the solution can be effectively precipitated as a recyclable hematite product, as opposed to forming the conventional hazardous jarosite residue, even under conditions where the potassium ion concentration reaches levels as high as 4 g/L. The composition of iron removal residue can be effectively controlled by adjusting the Na^+^/K^+^ concentration ratio in the simulated solution. Through the introduction of a substantial quantity of sodium ions, the collision frequency between sodium ions and ferric ions is heightened, while the collision frequency between potassium ions and ferric ions is diminished within the effective space, which promotes the formation of Na-jarosite. The decomposition transformation of Na-jarosite (32.64 kJ and 7.25 eV) is more energetically advantageous compared to that of K-jarosite (61.07 kJ and 15.31 eV). Driven by the advantages of thermodynamics and molecular dynamics, jarosite is transformed into recyclable hematite products. The verification experiment results in SZOP leaching solution showed that the iron content in the residues is above 58%, the sulfur content is below 4%, the zinc content is below 1%, and the total iron concentration in the supernatant is about 4 g/L, reaching the production index of the smelting industry. Our work can provide a new scheme for treating iron-containing solutions and wastewater in the chemical industry and metallurgy fields.

## Data Availability

The datasets analysed during the current study are available from the corresponding author on reasonable request.
